# *Helicobacter pylori* moderates the association between 5-MTHF concentration and cognitive function in older adults

**DOI:** 10.1371/journal.pone.0190475

**Published:** 2018-01-24

**Authors:** Andrew N. Berrett, Shawn D. Gale, Lance D. Erickson, Bruce L. Brown, Dawson W. Hedges

**Affiliations:** 1 Department of Psychology, Brigham Young University, Provo, Utah, United States of America; 2 The Neuroscience Center, Brigham Young University, Provo, Utah, United States of America; 3 Department of Sociology, Brigham Young University, Provo, Utah, United States of America; National Cancer Center, JAPAN

## Abstract

**Objective:**

To explore potential interactions between folate-cycle factors and *Helicobacter pylori* seropositivity in the prediction of cognitive function.

**Methods:**

We used data obtained from the 1999–2000 continuous National Health and Nutrition Examination Survey produced by the United States’ Centers for Disease Control and Prevention. Using Ordinary Least Squares regression, we tested for associations between multiple folate-cycle factors, *Helicobacter pylori* seropositivity, and cognitive function assessed by the digit symbol coding subtest of the Wechsler Adult Intelligence Scale-III. We then tested for interactions between each of the folate-cycle factors and *Helicobacter pylori* in the prediction of cognitive function.

**Results:**

Although *Helicobacter pylori* seropositivity, 5-methyltetrahydrofolate, vitamin B-12, and homocysteine were not associated with performance on the digit symbol coding task, *Helicobacter pylori* seropositivity interacted with 5-methyltetrahydrofolate concentration to predict performance on the digit symbol coding task. The *Helicobacter pylori* seropositive group performed worse on the digit symbol coding task as 5-methyltetrahydrofolate concentration decreased.

**Conclusion:**

The interaction between *Helicobacter pylori* seropositivity and reduced folate-cycle factor 5-methyltetrahydrofolate might impair aspects of cognitive function.

## Introduction

*Helicobacter pylori* (*H*. *pylori*) is a Gram-negative bacterium that resides in the gastrointestinal tract of a significant portion of the worldwide population [1]. The bacterium selectively inhabits the gastric epithelium of compatible hosts to initiate a persistent, latent infection. The release of urease, an ammonia-rich substance, allows *H*. *pylori* to safely reside in the highly acidic gastric environment [2]. Following infection, gastritis may occur and persist until successful treatment [3]. Infection with *H*. *pylori* has been associated with impaired or altered metabolism of nutrients such as folate and iron [4–6]. In addition, the presence of *H*. *pylori* in the gut initiates an ongoing inflammatory response with regional and systemic effects. Indeed, interactions between *H*. *pylori* and both folate and biomarkers of inflammation might predict cognitive function [7]. However, the degree to which *H*. *pylori* directly affects cognitive function [6, 8, 9] and the specific mechanisms by which *H*. *pylori* influences cognitive function remain unclear, including situations in which more than one mechanism might impair folate metabolism or inflammation.

Apart from *H*. *pylori* seropositivity, genetic variants might impair folate metabolism and consequent cognitive function. In particular, polymorphisms of the enzyme methylenetetrahydrofolate reductase (MTHFR), a key regulatory factor in the conversion of homocysteine to methionine, have been associated with cognitive deficits [10–12]. The MTHFR enzyme is responsible for the conversion of 5,10-methylenetetrahydrofolate to 5-methyltetrahydrofolate (5-MTHF) and the eventual donation of a methyl group to vitamin B-12. A co-factor in the folate cycle, vitamin B-12 then uses the methyl group in the conversion of homocysteine to methionine. Although several identified polymorphisms can affect the MTHFR enzyme, the C677T polymorphism is considered to be one of the most common. The prevalence of the C677T MTHFR mutation is variable and depends heavily upon ethnicity [13]. However, previous reports suggest that approximately 45% of the U.S. population may be CC homozygous (the normal variant), 45% CT heterozygous, and 10% TT homozygous [12, 14, 15]. This polymorphism results in reduced thermostability and efficiency of the MTHFR enzyme. In fact, one report suggested that MTHFR efficiency might be reduced by upwards of 40 to 50 percent in homozygous mutants [16]. Impaired MTHFR efficiency results in a stepwise decrease in 5-MTHF availability and a subsequent elevation of homocysteine, which might negatively affect cognitive function [17].

Although *Helicobacter pylori* seropositivity and MTHFR mutation likely affect folate metabolism and cognitive function by different mechanisms, they are not necessarily mutually exclusive of one another. An individual could be *H*. *pylori* seropositive and heterozygous or homozygous for the C677T MTHFR polymorphism. Indeed, with the relatively high prevalence of both *H*. *pylori* infection and MTHFR mutation, it is possible for both factors could interact to influence folate metabolism in a significant portion of the population. Since both MTHFR mutation and *H*. *pylori* infection negatively influence the same biochemical pathway, an interaction between both factors could impair folate metabolism and, subsequently, cognitive function beyond the effects of each factor alone. With this in mind, we considered whether interactions between *H*. *pylori* seropositivity and MTHFR polymorphisms are associated with cognitive function in older adults.

We used the continuous 1999–2000 National Health and Nutrition Examination Survey (NHANES) to obtain data for serum concentrations of 5-MTHF (a reduced form of folate and the direct product of the MTHFR enzyme), vitamin B-12, and homocysteine. Each of these folate-cycle factors is involved with folate metabolism, and 5-MTHF, in particular, provides a unique estimate of MTHFR efficiency in that low 5-MTHF concentrations suggest poor MTHFR function. Though genetic data were available in the NHANES data sets, access to the data is restricted and requires funding to access. Therefore, due to the direct relationship between 5-MTHF concentration and MTHFR function, we determined to use serum 5-MTHF concentration as a reasonable alternative. We hypothesized that reduced concentrations of serum 5-MTHF or vitamin B-12 or an increased concentration of homocysteine would be associated with worse cognitive function and that *H*. *pylori* seropositivity would moderate these associations.

## Materials and methods

### Study sample

The 1999–2000 continuous NHANES recruited 9,965 participants and used a variety of eligibility criteria to screen subsamples for data collection of the various measures we included in this study. The most restrictive criterion, for instance, was for the cognitive assessments, where only respondents aged 60 years or older were eligible. After taking into account eligibility for each of the study measures, a sample of 1,354 remained. We excluded three of these participants from the analyses because their elevated measures of homocysteine or folate suggested the possibility that the respondents were ill. The final analytic sample included 1,351 participants.

### Helicobacter pylori

CDC laboratory technicians determined seropositivity for *H*. *pylori* using an enzyme-linked immunosorbent assay kit from Wampole Laboratories (Wampole Laboratories, Division of Carter Wallace, Inc., Cranbury, NJ). The process requires the combination of participant serum with *H*. *pylori* specific antigens, goat anti-human IgG conjugated with horseradish peroxidase, and chromogen substrate serum. The result is a color change proportional to the concentration of *H*. *pylori* antibody in the participant’s serum that can be interpreted by a spectrophotometer. The 1999–2000 continuous NHANES did not publish raw IgG results as part of the 1999–2000 NHANES dataset, considering instead values less than .90 as negative for infection, values between .91 and 1.09 as equivocal, and values above 1.10 as positive for infection [18]. We treated subjects with equivocal results as negative for the purposes of these analyses. Importantly, positive IgG results do not necessarily indicate current *H*. *pylori* infection. For example, an individual previously infected with *H*. *pylori* but who has since eliminated it from the body can still test positive for *H*. *pylori* as IgG antibodies will remain after the infection. Additional details on this assay and its performance characteristics specific for U.S. adults in the 1999–2000 NHANES survey are available in NHANES documentation [18].

### 5-Methyltetrahydrofolate

5-MTHF (ng/mL) serum concentration was determined from stored sera specimens using an affinity/high performance liquid chromatography system. Calculation of 5-MTHF concentration was achieved by comparison to external and internal standards of known concentration [19]. We log transformed the 5-MTHF variable to adjust for a positive skew in the data.

### Vitamin B-12

Serum vitamin B-12 (pg/mL) concentrations were determined through the use of a Quantaphase II Folate/vitamin B-12 radioassay kit provided by Bio-Rad Laboratories [20]. Following preparation, labelled and unlabelled folate and vitamin B-12 compete for binding sites. Unbound folate and vitamin B-12 are discarded while bound substrate collects in pellet form. Radioactivity from the pellets is measured and compared to a standard curve to determine the concentration of vitamin B-12 in the participant’s blood. We log transformed the vitamin B-12 variable to adjust for a positive skew.

### Homocysteine

Homocysteine concentration (μmol/L) was determined through the use of a fluorescence polarization immunoassay (FPIA) from Abbott Diagnostics. The assay is performed automatically through the use of the Abbott IMX Immunoassay Analyzer and involves the reduction of homocysteine to S-adenosylhomocysteine (SAH), which is subsequently exposed to a specific monoclonal antibody and tracer. Total plasma homocysteine concentrations are calculated by the use of a machine-stored calibration curve [21]. We also log transformed homocysteine to account for a positive skew.

### Cognitive function

Survey participants 60 years or older completed the digit symbol coding (DSC) subtest of the Wechsler Adult Intelligence Scale–Third Edition [22]. Generally considered a measure of cognitive processing speed [23], the DSC is a timed test requiring participants to match symbols with their corresponding numbers as outlined by a key placed at the top of the form. Beyond processing speed, the DSC also requires sufficient ability in memory, attention, executive function, and visuomotor coordination in order to perform well in the task. Therefore, this task is a useful measure of overall cognitive ability and has been used as such in various psychiatric or neurologic populations [24–26]. In this study, the score on the DSC was the number of correctly completed matches. Participants with physical limitations such as blindness or those who could not successfully complete an initial practice phase did not participate in this test. Additionally, the 1999–2000 continuous NHANES did not report data from participants who stopped the test before the allotted time [27].

### Covariates

We included a number of covariates in our analyses to control for potential confounding. Categorical covariates included sex, race-ethnicity, education, tobacco-smoking status, gastrointestinal conditions, and food security. The race-ethnicity variable included categories for non-Hispanic whites, non-Hispanic blacks, Mexican Americans, and other ethnicities as suggested by NHANES documentation [28]. The education variable divided participants into groups based on whether they had a less than high-school, high-school, or more than high-school education at the time of the survey. Tobacco-smoking status categorized subjects into three groups: those who had never smoked tobacco, those who had smoked at least 100 cigarettes within their lifetime but who were no longer smokers, and those who had smoked at least 100 cigarettes in their lifetime and who were current smokers. Subjects were asked to indicate if they had previously experienced ulcers, colon cancer, rectal cancer, or stomach cancer at any point in their lives. Finally, to control for potential malnutrition, we included a food-security variable that gives four response options ranging from fully secure to insecure with hunger. Continuous covariates were the poverty-to-income ratio PIR, age, number of dietary supplements taken daily, and systolic blood pressure. PIR is the ratio between household income and the federally established poverty level at the time of the survey. Values above 1 are above the poverty threshold whereas values below 1 are in poverty. Age was recorded in years. NHANES top codes ages to 85 years; therefore, the reported age range in the data is 60 to 85 years. Daily supplement use data provides a general control for unnaturally high or low concentrations of key vitamins and minerals. Finally, especially in elderly populations, elevated blood pressure can be an indicator of increased risk for vascular disorders that could affect brain health and function [29, 30].

### Statistical analysis

We used Stata release 14.1 for all statistical analyses [31] and did all analyses using Stata’s *svy* command prefix, which allows for the inclusion of sampling weights to adjust parameter estimates to be representative of the U.S. civilian non-institutionalized population. In addition, the *svy* prefix allows for the inclusion of strata and cluster variables, which adjust standard-error estimates to account for the complex-sampling design of NHANES datasets.

We treated missing data with multiple imputation using chained equations. The chained equations approach allows the distribution of variables to be modelled appropriately in the imputation phase (e.g., continuous variables imputed using Ordinary Least Squares regression (OLS), dichotomous variables imputed using logistic regression, etc.) [32]. To allow imputed values to be sensitive to interactions of *H*. *pylori* with the folate-cycle factors, we estimated separate imputation models for respondents who were seropositive and seronegative for *H*. *pylori*. We created twenty imputed datasets because the variable with the highest amount of missing data was below 20 percent [33]. The imputed datasets were separated by 400 iterations because diagnostics indicated that the imputation model converged before that point [32]. We conducted all analyses using Stata’s *mi estimate* prefix, which uses Rubin’s rules to combine the results from each imputed dataset into a single set of estimates [34].

We used OLS regression to test whether individual folate-cycle factors (5-MTHF, homocysteine, and vitamin B-12) or *H*. *pylori* infection were independently associated with cognitive function. We then tested for potential interactions between *H*. *pylori* infection and serum concentrations of 5-MTHF, homocysteine, and vitamin B-12 in predicting cognitive function as assessed by the number of correct responses on the DSC. All analyses included sex, race-ethnicity, education, tobacco-smoking status, history of gastrointestinal conditions, food security, PIR, age, number of dietary supplements taken daily, and systolic blood pressure as controls.

## Results

Forty-one percent of the sample was seropositive for *H*. *pylori*, 45% was male, and the large majority (80%) was non-Hispanic white. The average age was 70 years. Table 1 shows other sample characteristics.

**Table 1:** Weighted summary statistics of U.S. adults 60 to 85-year-olds, National Health and Nutrition Examination Survey 1999–2000.

	Mean or Frequency
DSC Number Correct	45.38 (.90)
*H*. *pylori* Seropositive	548 (40.55%)
Folate Cycle Factors	
5-MTHF (ng/mL)	22.90 (.37)
Vitamin B-12 (pg/mL)	555.91 (11.42)
Homocysteine (μmol/L)	9.80 (.12)
Age	70.40 (.38)
Poverty-to-income Ratio	2.61 (.12)
Dietary Supplement Count	1.88 (.13)
Systolic Blood Pressure	139.74 (.86)
Male	602 (44.54%)
Education	
Less than High School	472 (34.95%)
High School	386 (28.60%)
More than High School	492 (36.45%)
Race-ethnicity	
Non-Hispanic white	1084 (80.21%)
Non-Hispanic black	103 (7.59%)
Mexican American	40 (2.95%)
Other	125 (9.25%)
Cigarette Smoking	
Never smoked	641 (47.41%)
Former smoker	535 (39.58%)
Current smoker	176 (13.02%)
Gastrointestinal Conditions	
Ulcers	216 (15.96%)
Cancer^a^	22 (1.60%)
Food Security	
Fully secure	1242 (91.93%)
Marginally secure	46 (3.38%)
Insecure without hunger	41 (3.05%)
Insecure with hunger	22 (1.64%)

^a^Including stomach, rectal, and colon cancers.

Note: Means (SE) or frequencies (%) are reported for each variable.

Abbreviations: DSC = Digit symbol coding, *H*. *pylori = Helicobacter pylori*, SE = Standard error

N = 1,351

There were no associations between 5-MTHF concentration, homocysteine concentration, vitamin B-12 concentration, and *H*. *pylori* seropositivity and DSC performance (Table 2).

**Table 2:** Linear regression analysis of folate cycle factor concentrations and *Helicobacter pylori* infection status predicting cognitive function in U.S. 60 to 85-year-olds.

	DSC Correct		
	β	95% CI	*p*
Model 1: 5-MTHF	1.73	[-.03, 3.48]	.053
Model 2: Vitamin B-12	-1.05	[-2.92, .83]	.240
Model 3: Homocysteine	-2.69	[-5.83, .44]	.085
Model 4: *H*. *pylori*	-.53	[-3.07, 2.01]	.656

Note: Age, sex, education, race-ethnicity, poverty-to-income ratio, smoking status, dietary supplement use, systolic blood pressure, food security, and prior history of ulcers, stomach cancer, colon cancer, and rectal cancer included as controls in all models.

Abbreviations: DSC = Digit Symbol Coding; CI = Confidence interval; MTHF = Methyltetrahydrofolate; *H*. *pylori* = *Helicobacter pylori*

N = 1,351

**Fig 1:**
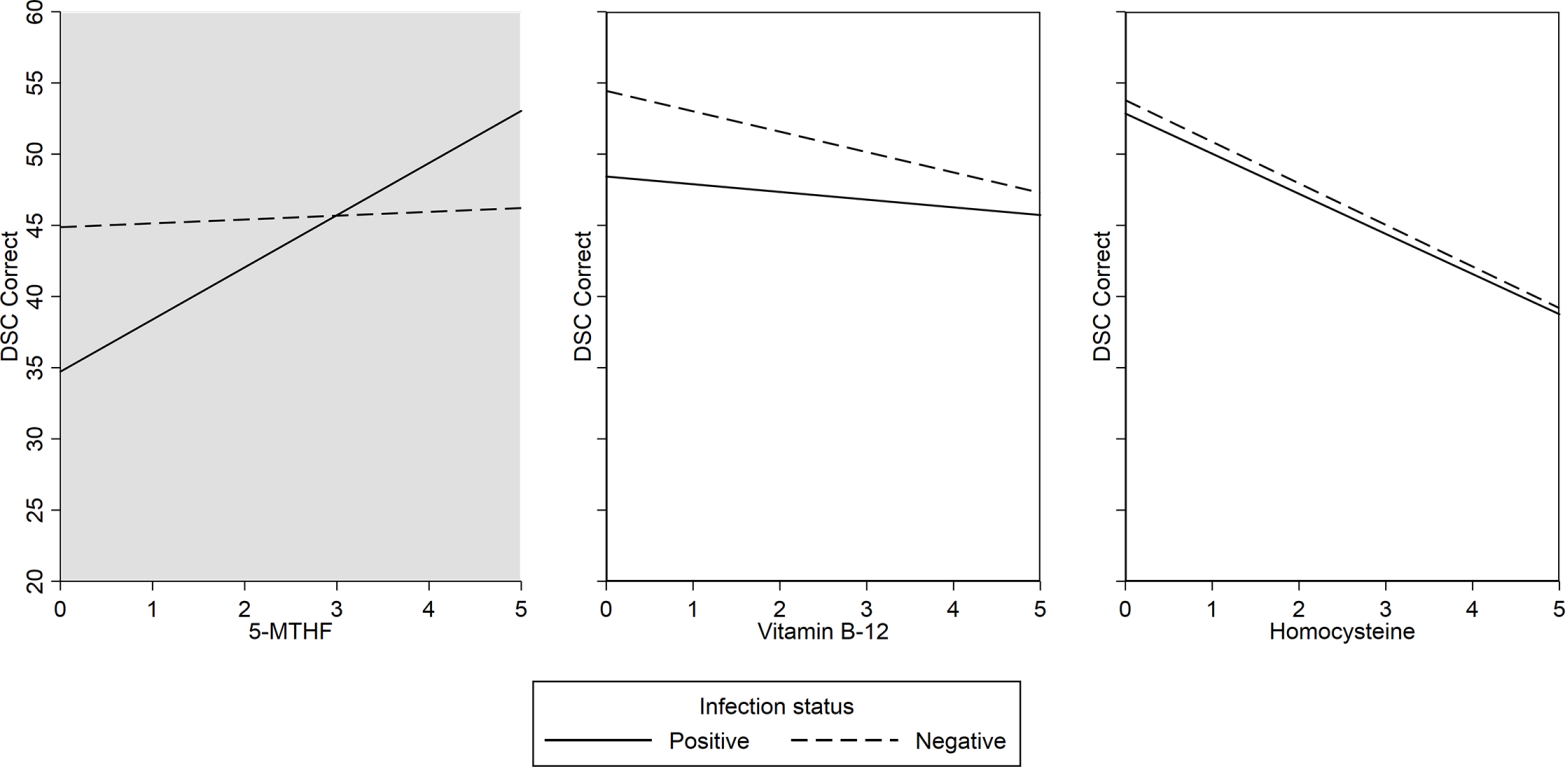
Number of correct DSC responses predicted from interactions of *H*. *pylori* infection status and multiple folate-cycle factors in adults aged 60 to 85 years. Note: Shaded plots indicate statistical significance at the *p* = .05 level.

To determine whether *H*. *pylori* seropositivity moderates the associations between any of the folate-cycle factors and cognitive function, we did three separate analyses testing for interactions between *H*. *pylori* and each of the folate-cycle factors in the prediction of DSC performance. In these analyses, only 5-MTHF significantly interacted with *H*. *pylori* seropositivity to predict DSC performance (β = 3.35 [.01, 6.90]; *p* = .049) (Table 3, Model 1). Specifically, with less available serum 5-MTHF, performance on the DSC progressively worsened in subjects seropositive for *H*. *pylori*, suggesting that subjects seropositive for *H*. *pylori* and who simultaneously also had less bioavailability of 5-MTHF at the time of examination performed worse on the DSC when compared to uninfected subjects (Fig 1). There were no significant interactions between *H*. *pylori* infection status and vitamin B-12 or homocysteine (Table 3). To account for potential confounding interactions between the folate cycle factors explored in this study, we repeated each interaction analysis with the other folate factors added as controls. For example, we repeated the test for an interaction effect between *H*. *pylori* and 5-MTHF while controlling for vitamin B-12 and homocysteine as well as the other controls previously incorporated. These analyses produced nearly identical results to our original estimations (not reported).

**Table 3:** Two-way interaction effects of folate cycle factor concentrations and Helicobacter pylori infection status predicting cognitive function in U.S. 60 to 85-year-olds.

	DSC Correct		
	β	95% CI	*p*
Model 1:			* *
5-MTHF	.31	[-1.93,2.55]	.768
*H*. *pylori*	-10.00	[-20.30, .31]	.056
*H*. *pylori* x 5-MTHF	3.35	[.01, 6.90]	.049
Model 2:			
Vitamin B-12	-1.44	[-4.14, 1.27]	.264
*H*. *pylori*	-5.97	[-35.62, 23.67]	.666
*H*. *pylori* x Vitamin B-12	.89	[-3.95, 5.73]	.694
Model 3:			
Homocysteine	-3.01	[-6.37, .35]	.074
*H*. *pylori*	-2.29	[-15.81, 11.23]	.714
*H*. *pylori* x Homocysteine	.78	[-5.29, 6.84]	.781

Note: Age, sex, education, race-ethnicity, poverty-to-income ratio, smoking status, dietary supplement use, systolic blood pressure, food security, and prior history of ulcers, stomach cancer, colon cancer, and rectal cancer included as controls in all models.

Abbreviations: DSC = Digit Symbol Coding; CI = Confidence interval; MTHF = Methyltetrahydrofolate; *H*. *pylori = Helicobacter pylori*

N = 1,351

## Discussion

The main results of this study show that in older US adults, reduced 5-MTHF availability and *H*. *pylori* seropositivity interact to reduce cognitive function assessed with the DSC. In contrast, there were no main effects for 5-MTHF, vitamin B-12, homocysteine, and *H*. *pylori* seropositivity in predicting performance on the DST, although there was weak support for an association between higher 5-MTHF concentration and better performance on the DSC with a *p* value of 0.053. Neither vitamin B-12 nor homocysteine interacted with *H*. *pylori* seropositivity to predict DSC performance.

In contrast to other studies, we did not find a statistically significant association between homocysteine and cognitive function. In a study of community dwelling elderly subjects in Dublin, Chin et al. [35] found that homocysteine was inversely associated with cognitive function. However, the average age in the Chin et al. [35] study was higher than in our study (75 years vs. 70 years), as was the average homocysteine level (13.9 μmol/L vs. 9.75 μmol/L). Differences in age and average homocysteine concentration might account for some of the differences between our findings and those of Chin et al. [35]. Some evidence suggests that higher homocysteine levels may be more closely associated with certain cognitive domains, such as executive functioning and processing speed than with other cognitive domains [36, 37]. The limited cognitive testing available in the NHANES dataset we used might have precluded our finding an association between homocysteine concentration and cognitive function, in that we assessed cognitive function with only the DSC [38].

In testing for an association between *H*. *pylori* seropositivity and cognitive function, we found no significant differences between seropositive and seronegative groups, adding to the already mixed literature on whether *H*. *pylori* seropositivity alone is associated with cognitive functioning [7–9, 39–41]. Despite the uncertainty about the independent effects of *H*. *pylori* on cognitive function, our findings suggest that *H*. *pylori* seropositivity might contribute to other processes or conditions in which cognitive function might already be susceptible, such as with reduced 5-MTHF availability affecting key pathways related to the metabolism of important nutrients like folate. Our findings suggest that *H*. *pylori* seropositivity might be an important moderating factor in determining cognitive function related to 5-MTHF availability.

The exact mechanism by which *H*. *pylori* seropositivity and reduced 5-MTHF availability might interact to influence folate metabolism is unclear. It is possible that the chronic gastritis associated with *H*. *pylori* infection could lead to impaired folate or vitamin B-12 uptake or both [42]. In either case, polymorphisms in enzymes related to folate metabolism could exacerbate the already decreased folate concentration, resulting in fewer available folate metabolites and less efficient enzymatic processes. Indeed, efficiency of the MTHFR enzyme is reduced by about 40 to 50% in TT homozygous individuals [16]. Therefore, reduced enzymatic activity due to an MTHFR polymorphism coupled with reduced serum folate availability due to *H*. *pylori* infection could result in less available 5-MTHF than in either situation alone.

Reduced folate and/or 5-MTHF availability are associated with several clinical conditions including developmental defects and various psychological and cognitive outcomes. For example, embryos developing in an environment with reduced availability of folate are at greater risk of neural tube defects and spontaneous abortion [43]. In addition, higher prevalences of both autism spectrum disorder and Down syndrome are associated with MTHFR polymorphisms [44–46]. These developmental risks motivate the recommendation for pregnant mothers to regularly supplement with folate throughout pregnancy. While proton-pump inhibitors and antibiotics can treat *H*. *pylori* infection [47] and folate supplementation can improve low concentrations of folate metabolites [48], it is unclear whether these solutions are sufficient to overcome the combined deleterious effects of *H*. *pylori* infection and reduced 5-MTHF availability. It is possible that even with regular folate supplementation, a pregnant woman infected with *H*. *pylori* and homozygous or heterozygous for an MTHFR polymorphism could still have low concentrations of available folate metabolites. In this case, a fetus could remain at risk of developmental issues despite the mother following the traditional clinical recommendations.

Beyond brain development, MTHFR polymorphisms are also potentially associated with Parkinson’s disease, Alzheimer’s disease, migraine, and depression [11, 49–52]. Further, multiple reports show associations between folate deficiency and each of these disorders in older adults [53–55]. Similar to the case of pregnant women, an interaction between 5-MTHF concentration and *H*. *pylori* infection might accelerate or enhance the associations between age-related folate deficiency and the onset of neurological or cognitive disorders. Also, it is unknown whether folate supplementation is sufficient to overcome the potential effects of an interaction between an MTHFR polymorphism and *H*. *pylori* infection in older adults. With the number of associations between deficiencies in folate metabolism and various brain disorders, our findings indicate a need for continued investigation into the compounding effects of simultaneous MTHFR mutation and *H*. *pylori* infection and in in determining whether folate supplementation is sufficient to overcome the effects of this interaction.

This study has a number of limitations and strengths. Due to the cross-sectional nature of the NHANES datasets, we were unable to determine the time of *H*. *pylori* infection, precluding understanding how infection duration might affect cognition. Also, serum 5-MTHF concentration or concentrations of other folate-cycle factors are not direct indicators of MTHFR polymorphisms as only genetic testing can determine MTHFR genotype. Although the 1999–2000 NHANES dataset does contain MTHFR genotype data, the genotype data are restricted and require funding to access. In a future study, we intend to obtain the restricted NHANES genetic data to analyse directly associations between MTHFR variants, infections, and cognitive function. Another factor to consider in interpreting these findings is that the sample consisted of adults aged 60 to 85 years. The interaction betweeen5-MTHF and *H*. *pylori* seropositivity in predicting cognitive function might be different in other age groups. In addition, the DSC was the only measure of cognitive function available in the 1999–2000 NHANES dataset. Future studies could use additional measures of cognitive function to ensure the findings of this study are not specific to the DSC. Strengths of the study, however, included the large sample size and good generalizability to the US population due to the complex multi-stage design the NHANES surveys use. Also, the use of multiple folate-cycle factors allowed for better identification of the relationships between *H*. *pylori* seropositivity and folate-cycle factors in predicting cognitive function.

In conclusion, this study presents preliminary evidence of a potential interaction between the availability of serum 5-MTHF and *H*. *pylori* seropositivity in predicting decreased cognitive function in US adults aged 60 years and older. This finding might be especially relevant to vulnerable groups such as pregnant women and the elderly.
